# Supplementary material for: An open-source framework for large-scale, flexible evaluation of biomedical text mining systems
Source: J Biomed Discov Collab. 2008 Jan 29;3:1. doi: 10.1186/1747-5333-3-1 (PMC2276192; doi:10.1186/1747-5333-3-1)
Supplement: Additional file 1 — Evaluation platform source code. The additional file contains the software evaluation framework discussed in this paper. The most current release can be downloaded from SourceForge.net [1]. See the accompanying README file for instructions on installation and use. Also included in the distribution is a collection of UIMA wrappers for some commonly used BioNLP tools and annotated corpora. [file 1747-5333-3-1-S1.zip › bionlp_uima_repository-1.0.1/docs/api/deprecated-list.html]

Deprecated List (Center for Computational Pharmacology Util/UIMA API)


|  |  |  |
| --- | --- | --- |
| **Overview** \| Package \| Class \| **Tree** \| **Deprecated** \| **Index** \| **Help** | |  |
| PREV   NEXT | **FRAMES**    **NO FRAMES**     **All Classes** |  |


---


## **Deprecated API**


---

**Contents**

- Deprecated Methods- Deprecated Constructors

**Deprecated Methods**

edu.uchsc.ccp.util.nlp.document.GenericDocument.addSecondaryDocumentID(String)

edu.uchsc.ccp.util.nlp.document.GenericDocument.getAbstractText()

edu.uchsc.ccp.util.nlp.mention.ClassMention.getComplexSlotMentionByName(String)

edu.uchsc.ccp.util.nlp.annotation.TextAnnotation.getHashKey()

edu.uchsc.ccp.util.nlp.mention.SlotMention.getHashKey()

edu.uchsc.ccp.util.nlp.document.GenericDocument.getSecondaryDocumentIDs()

edu.uchsc.ccp.util.nlp.mention.ClassMention.getSlotMentionByName(String)

edu.uchsc.ccp.util.nlp.document.GenericDocument.getTitleText()

edu.uchsc.ccp.util.nlp.document.GenericDocument.setAbstractText(String)

edu.uchsc.ccp.util.nlp.annotation.TextAnnotation.setAnnotatorID(int)

edu.uchsc.ccp.util.nlp.mention.Mention.setMentionName(String)

edu.uchsc.ccp.util.nlp.document.GenericDocument.setSecondaryDocumentIDs(List)

edu.uchsc.ccp.util.nlp.document.GenericDocument.setTitleText(String)

**Deprecated Constructors**

edu.uchsc.ccp.util.nlp.mention.ClassMention()

edu.uchsc.ccp.util.nlp.mention.ComplexSlotMention()

edu.uchsc.ccp.util.nlp.mention.Mention()

edu.uchsc.ccp.util.nlp.mention.SlotMention()

edu.uchsc.ccp.util.nlp.annotation.TextAnnotation()

---


|  |  |  |
| --- | --- | --- |
| **Overview** \| Package \| Class \| **Tree** \| **Deprecated** \| **Index** \| **Help** | |  |
| PREV   NEXT | **FRAMES**    **NO FRAMES**     **All Classes** |  |


---
